# Supplementary material for: Biochemical Composition and Biological Activities of Various Population of Brassica tournefortii Growing Wild in Tunisia
Source: Plants (Basel). 2022 Dec 6;11(23):3393. doi: 10.3390/plants11233393 (PMC9739365; doi:10.3390/plants11233393)
Supplement: Supplementary file 1 [file plants-11-03393-s001.zip › plants-1999788-supplementary.pdf]

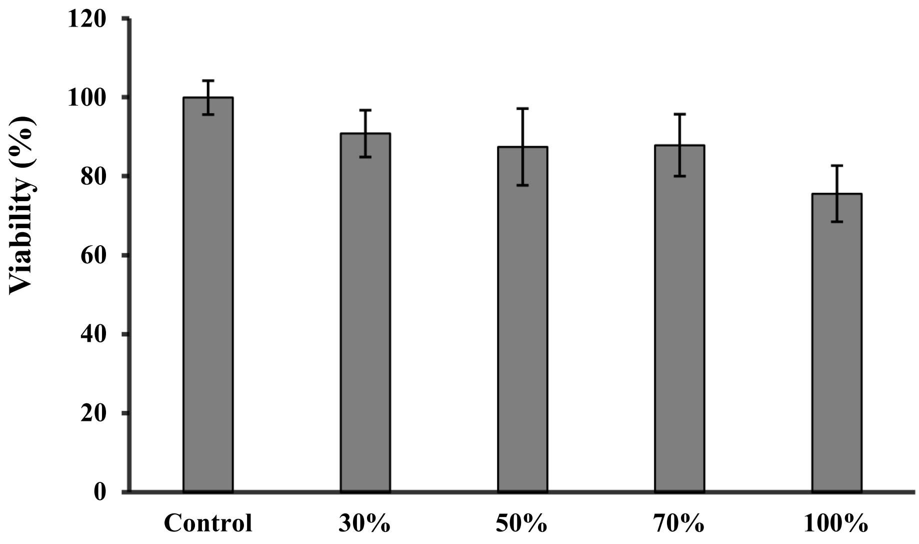

	Control	30%	50%	70%	100%
moy	100	90.8795	87.515723	87,9493334	75.660101
ecart	4.292790989	5.94133	9.7138925	7.83685243	7.1095741

Figure S1. Cell viability in 70% ethanol. MTT results showing the cell viability (%) for the K-562 cell line incubated with 70% ethanol compared to that one of the un-treated cells (control).

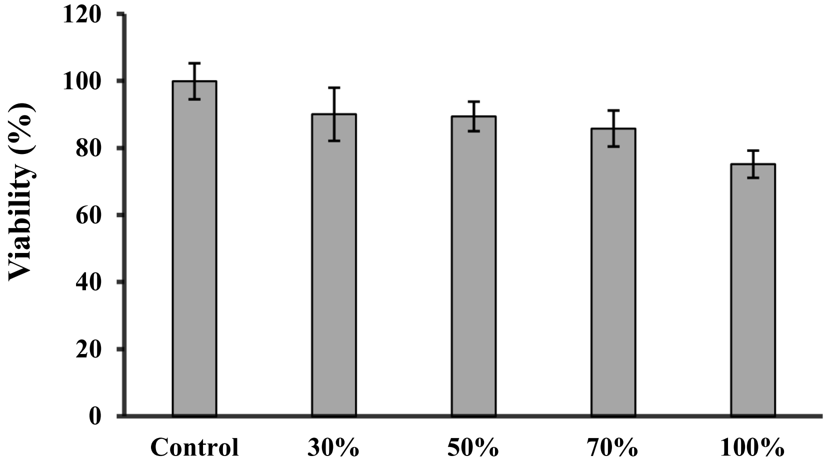

	Control	30%	50%	70%	100%
moy	100	90.167	89.534051	85.9165458	75.266838
ecart	5.379512647	7.9261	4.4082868	5.3616948	4.0648853

Figure S2. Cell viability in 70% ethanol extract. MTT results showing the cell viability (%) for the Caco-2 cell line incubated with 70% ethanol extract, compared to that one of the un-treated cells (control).
